# Aging and the Detection of Visual Errors in Scenes

**DOI:** 10.4061/2011/984694

**Published:** 2011-10-04

**Authors:** Lori E. James, Toni M. Kooy

**Affiliations:** Psychology Department, University of Colorado Colorado Springs, Colorado Springs, CO 80918, USA

## Abstract

Young and older adults performed a visual error detection task in two experiments. In Experiment 1, errors and anomalies were embedded in large, complex visual scenes, and participants were to find them and describe the nature of the identified problems. Young adults found more errors than older adults, a finding unrelated to age differences in near visual acuity or time constraints. Experiment 2 replicated the age difference in error detection using simplified visual scenes containing fewer errors. Results are interpreted as reflecting older adults' decreased ability to form representations for novel information, even though the task did not require the creation of new episodic memories.

## 1. Aging and the Detection of Visual Errors in Scenes

Relatively little research to date has tested age-related changes in error detection. Among the existing studies, some have examined young and older individuals' abilities to identify errors that they committed themselves (e.g., [1–3]). While these studies provide very naturalistic tests of error detection, interpretation of the results is complicated because there are often age differences in error production. 

Other studies have tested the effects of aging on the detection of experimenter-provided errors in written language. Zabrucky and Moore [4] found no age differences in detecting different types of errors in text (including nonsense words, false information, and inconsistent information). Shafto [5] found that spelling errors in text were detected equally often by young and older adults, but that older adults were impaired relative to young adults in detecting errors of meaning (e.g., the word “sun” where the word “moon” is actually appropriate), apparently because the semantic context leads to miscomprehension of the presented word. MacKay et al. [6] found equivalent performance for young and older adults on a task requiring the detection of misspelled words. Shafto [7] demonstrated that older adults actually outperformed young adults on misspelling detection, a finding driven by older adults' better ability to identify erroneous spellings of low-frequency items. Such tasks tap language processes, and it is not clear whether preserved error detection per se or language comprehension processes (which have been shown to be relatively stable in older adulthood; see, e.g., [8, 9]) account for the findings.

Additional research has tested the detection of errors or anomalies introduced by an experimenter using nonlinguistic stimuli. Simons and Levin [10] tested “change blindness” by measuring how likely an observer was to notice that his or her conversational partner had been surreptitiously replaced midconversation. Their nonyoung participants (approximate ages 35–65) were more susceptible to change blindness than young participants (ages 20–35). A second experiment demonstrated that an outgroup effect was responsible for the increased change blindness in nonyoung participants (i.e., that the change in the young conversational partners went unnoticed by older participants because of different social group membership; e.g., [11]). However, the apparent age difference could also be interpreted as reduced error detection among adults over age 35, a possibility that was not tested. Subsequent research has revealed age-related declines in the detection of a changing segment within a pictured scene [12] and in an array of rectangles [13]. Costello et al. [14] also found that older adults were impaired relative to young adults in a visual change detection task, and that declines in perceptual speed were an important contributor to the obtained age difference. However, these studies all involve a memory component, because two nonsimultaneous representations must be compared in order to detect the error or change. New episodic memory formation is known to decline with aging (e.g., [15, 16]), making it impossible to conclude whether the observed age differences reflect changes in error detection per se or whether they result from age-related memory changes. The current research employed a task that tests identification of errors, independent of the need to form new episodic memories. 

The task we selected was adapted from a common childhood game: the detection of errors and anomalies within drawings of common scenes. Following the distinction made by Veiel et al. [13], our participants performed a visual detection task that was not purely a visual search task (which has been shown in many previous experiments to decline with age; e.g., [17, 18]). While visual search tasks involve scanning a visual display to respond to a specific item, our task had multiple, nonspecified targets. Additionally, unlike visual search tasks, per-item response time was not measured and the task was not designed to induce time pressure. We also reasoned that the selected task would be familiar to participants and quite engaging, as it is sometimes included in magazines for entertainment. 

Within the literature on cognitive aging, older adults' decreased ability to form new representations that integrate diverse pieces of information has been suggested as the primary cause of age differences in memory performance. For example, age-related disruption of associative or binding mechanisms has been suggested as a critical determinant of older adults' cognitive performance within the transmission deficit hypothesis (TDH; [16]), the associative deficit hypothesis [19], and other frameworks (e.g., [20, 21]). These hypotheses generally focus on the formation of new, long-term, episodic memories for associated information, but the TDH in particular has accounted for age-related changes in novel language processing (e.g., [6, 22]) and has been applied to perceptual processes as well (e.g., [23, 24]). Specifically, the TDH suggests that older adults' deficits in forming novel associations should disrupt perception of novel visual information or novel arrangements of familiar visual information, in addition to the well-documented disruption of the formation of new episodic associative memories. 

MacKay and James [23] describe a series of processes necessary to successfully detect errors in visual scenes. ^1^ They propose that people must form a new representation of the error or anomaly by associating visual elements, and then compare that new representation with their preexisting internal representation of the nonerroneous object or situation from which it has been derived. Because the formation of internal representations for never-previously-encountered errors via binding is problematic for older adults, we predicted age-related declines in error detection.

## 2. Experiment  1

Participants inspected drawings of commonplace settings (e.g., a playground) containing many familiar items as well as errors and anomalies (e.g., a swing with a license plate for a seat; a gymnast wearing one high-heeled shoe). To successfully detect the errors, participants had to form new internal representations of what they viewed, and identify differences between the actual and expected content of the scenes (e.g., a traditional swing seat; a barefoot gymnast). We predicted that older adults would detect fewer errors than young adults. 

### 2.1. Method

#### 2.1.1. Participants

Eighteen young and 18 healthy older adults (characteristics presented in Table 1) provided their informed consent and completed the experiment. The groups had similar years of education, *t*(33) = 0.27, *P* > 0.79. Older adults' mean near vision score (averaged across scores for left and right eye for each participant) was worse than young adults', *t*(34) = 2.32, *P* < 0.05. Young adults received extra course credit and older adults were paid for their participation.

#### 2.1.2. Materials and Procedure

Four detailed, colorful drawings of complex but familiar scenes containing multiple errors and anomalies were selected from the *What's Wrong Here*? books designed for children. Each participant received an 11 × 14′′ color photocopy of a picture on which they were instructed to circle all errors, describing each error as they found it. Each participant held the picture in front of himself or herself, or placed it on the table at a comfortable viewing distance of his or her own choosing. The first drawing served as practice: a school cafeteria in which a boy is wearing only one shoe and a candy cane serves as a railing (among many other errors). The participant found and described as many errors as possible, and asked any questions about the procedure during the practice trial. Most participants asked no questions about the instructions, and the most common type of question was to indicate a potential problem they saw and to verify whether this should be considered an error. The experimenter also explained any errors that the participant failed to detect or describe. No participant appeared to be confused by task instructions, and performance on the practice trial indicated that each participant was familiar with how to perform the task. Three critical drawings were presented in the same randomly determined order, and participants were allowed up to 5 minutes to detect and describe errors in each picture. Picture 1 was a school gymnasium scene (with 26 errors), Picture 2 was a playground scene (with 12 errors), and Picture 3 was a scene at an outdoor ice skating rink (with 24 errors). Correctly identified errors were scored from audio tape recordings along with markings on the pictures. Time required to detect and describe all errors in each picture was collected from the recordings via stopwatch. 

Each participant completed an informed consent form, a demographics sheet, and the vision test prior to the experiment. The vision test was performed with corrective lenses for participants who wore them, and involved a standard near vision eye chart. Older adults also completed the Mini Mental Status Exam [25] and all achieved adequate scores for inclusion (minimum of 27 out of 30 correct).

### 2.2. Results and Discussion

A 2 (age group) × 3 (stimulus picture) ANOVA on percent of errors correctly detected indicated that young adults outperformed older adults, *F*(1,34) = 10.56, partial *η* ^2^ = 0.24, *P* < 0.01 (see Table 2). There was also a main effect of picture, *F*(2,68) = 35.88, partial *η* ^2^ = 0.51, *P* < 0.01, but no interaction of age and picture, *F* < 1, partial *η* ^2^ < 0.01, because young adults outperformed older adults to a similar degree for all three pictures. Independent groups *t-*tests confirmed the age difference for each picture separately, *t*(34) = 2.78, *P* < 0.01, *t*(34) = 3.29, *P* < 0.01, and *t*(34) = 2.64, *P* < 0.05. Whereas that analysis compared the percent of errors within each picture that were detected by each participant, a second analysis compared the percent of young and older participants who detected each error within each picture. This by-items 2 (age group) × 3 (stimulus picture) ANOVA indicated that young adults outperformed older adults, *F*(1,59) = 23.94, partial *η* ^2^ = 0.29, *P* < 0.01, but no main effect of picture, *F*(2,59) = 2.29, partial *η* ^2^ = 0.07, *P* > 0.05, and no interaction of age and picture, *F* < 1, partial *η* ^2^ < 0.01. Paired *t*-tests confirmed that a greater percent of young than older adults identified each error for each picture separately, *t*(25) = 3.46, *P* < 0.01, *t*(11) = 2.85, *P* < 0.05, and *t*(23) = 4.01, *P* < 0.01. 

Age differences in visual acuity are well documented (e.g., [26]), and our vision test results indicate that young adults had more accurate near vision than older adults. However, this cannot account for the age difference in error detection because vision scores did not correlate with percent of errors detected across all participants, *r*(36) = −0.25, *P* > 0.13, nor within young participants, *r*(18) = −0.21, *P* > 0.40, or older participants, *r*(18) = −0.06, *P* > 0.81. Results also provide evidence against speed-accuracy tradeoff as the source of age differences in error detection: older adults spent more time detecting and describing errors in each picture than young adults, although this age difference was not significant for any picture alone, each *P* > 0.15, nor for the total time of all pictures combined (young *M* = 415 s, SD = 197; older *M* = 466 s, SD = 217), *F* < 1, partial *η* ^2^ = 0.01. Neither age group approached the maximum total time allowed (900 s), indicating that time pressure did not cause the age difference in error detection. These data also indicate that participants in both age groups were motivated to find all errors because young and older adults spent a reasonably and similarly long time searching for errors in each picture.

Experiment 1 results support our prediction that older adults would detect fewer errors than young adults. Visual acuity did not correlate with number of errors detected, and there was no indication that older adults spent less time trying to find errors than did young adults, ruling out these explanations of the age difference. When measured on a task that does not engage episodic memory or language comprehension processes, older adults' error detection is inferior to young adults'.

## 3. Experiment 2

Although there was no evidence that visual acuity was related to age differences in error detection in Experiment 1, the large number of errors in each scene may have proved visually confusing or interfering for older participants, negatively impacting their performance. Indeed, older adults report increased difficulty with “cluttered visual scenes” [27, page 2], and it appears that with increased age, “perceptual processing of one stimulus is more likely to be distracted by the presentation of another stimulus” [28, page 416]. To examine the possibility that our Experiment 1 results resulted from the complexity of the visual scenes we used, we replicated the study using pictures containing fewer errors. The goal was to assess the generalizability of the age-related deficit in error detection, and we predicted an age-related difference favoring young adults as in Experiment 1.

### 3.1. Method

#### 3.1.1. Participants

Twenty young and 20 healthy older adults (see Table 1) participated. Five additional participants were tested but their data were not useable due to a tape-recorder malfunction. The groups had similar years of education, *t*(37) = 0.63, *P* > 0.53, and there was no age difference in average near vision score, *t*(38) = 1.34, *P* > 0.18. Participants provided informed consent and were compensated as in Experiment 1.

#### 3.1.2. Materials and Procedure

 Seven stimulus pictures were selected: some were subsections from illustrations in *What's Wrong Here*? (i.e., they contained errors) and others came from various children's books and did not initially contain errors. For a different purpose, each picture was altered so that there were two versions of it: one containing a small number of errors (2–5) and another with no errors. This manipulation was not relevant to the current results and is not discussed further (i.e., only data for pictures that contained errors are reported here). Pictures were approximately 6 × 8′′ in size. One picture served as practice, and two sets with three pictures each were created. The procedure was identical to Experiment 1: participants were to clearly indicate any identified errors or anomalies. Sessions were audio taped and error detection was scored from the recordings along with markings on the pictures. Informed consent, demographic information, visual acuity scores, and a measure of cognitive function were collected as in Experiment 1.

### 3.2. Results and Discussion

An independent groups *t-*test indicated that young adults detected more errors than older adults for all pictures combined, *t*(38) = 3.13, *P* < 0.01 (see Table 2). ^2^ A by-items analysis (using a paired *t*-test) also indicated that a greater percent of young than older adults identified each error across all pictures, *t*(18) = 4.13, *P* = 0.001. 

In this experiment, there was no significant age difference in visual acuity. Presumably, there would have been if participants had not been allowed to wear their corrective lenses, but our interest was in ensuring that participants could see the pictured information, so we did not measure uncorrected vision. Furthermore, vision scores did not correlate with percent of errors detected across all participants, *r*(40) = −0.17, *P* > 0.29, nor within young participants, *r*(20) = −0.02, *P* > 0.93, or older participants, *r*(20) = −0.13, *P* > 0.58. Experiment 2 results replicate Experiment 1, again showing that young adults detected more errors than older adults, even within simple, noncluttered pictures containing few errors.

## 4. General Discussion

Older adults detected fewer errors than young adults, whether the scenes were large and complex, containing many errors, or small and uncluttered, with few errors. This is an important extension of previous research on the detection of experimenter-introduced errors because the current task does not confound error detection with the requirement to form new episodic memories or with language comprehension ability. Importantly, we were able to rule out time pressure and age-related changes in visual acuity as explanations of the obtained age differences.

These results support the suggestion that age-related difficulty in forming new associations during a task without an episodic memory requirement impacts the domain of visual perception. Theoretical frameworks that posit binding or associative deficits to account for patterns of memory function in aging (e.g., [16, 19, 21]) are useful in accounting for the present results. To date, within this class of theories, only the TDH [16] has been developed to account for perceptual processes using binding mechanisms (e.g., [24]) as well as error detection (e.g., [2, 23]) while the other theories are focused on the formation of associations to explain only phenomena related to memory. Under the TDH, the age deficit in detecting experimenter-presented visual errors occurs because the first step in error detection is for the participant to form a novel representation of each error or anomaly by associating the presented visual components. In other words, age-related decrements in the ability to make novel associations for never previously encountered errors underlie the obtained age-related declines in error detection.

Other findings regarding visual perception in aging are consistent with an interpretation based on older adults' deficits in forming novel representations. For example, Basowitz and Korchin [29] found that young adults outperformed older adults in identifying objects from drawings that were partially obliterated. An analysis of incorrect responses in that study indicated that young adults provided incorrect labels that were similar to the actual objects they were attempting to identify (e.g., saying “bear” to a picture of a dog), whereas older adults gave responses that reflected fragmented or loosely organized representations (e.g., “a pile of rocks” or “clouds”), which possibly reflects age-related impairment in forming coherent representations of the fragmented objects. As another example, Soldan et al. [30] demonstrated age stability in priming of familiar visual objects but age-related declines in priming of novel visual objects. Under the theoretical account we suggest here, older adults in Soldan et al. were unable to speed their responses to a second presentation of the objects (i.e., they failed to demonstrate priming) because they had difficulty forming stable novel representations of the previously unfamiliar objects when they were initially presented. 

Other theoretical approaches to cognitive aging seem unlikely to account for the obtained age differences in error detection. For example, theories that posit that age-related general slowing impairs cognitive performance (e.g., [31, 32]) cannot explain the present results because older participants did not approach the maximum allotted time to perform the task, and there was no age difference in time to find and describe the errors in Experiment 1. The idea that sensory deficits underlie age differences in cognitive ability (e.g., [33]) also does not fit the current data because visual acuity did not correlate with error detection performance, and did not differ between young and older adults in Experiment 2. The inhibitory deficit hypothesis (e.g., [34]) might predict an effect of age on error detection ability because other elements within the scene could be distracting, and interfere with processing of an individual error. The challenge for the inhibitory deficit hypothesis is that it predicts that the more potentially interfering information (including errors) contained in a scene, the greater the disruption due to activation of irrelevant information, especially for older adults. Current results indicate age decrements for both simple (with as few as 2 errors) and complex scenes (with as many as 26 errors). However, a systematic test of age differences for pictures with varying numbers of errors is needed to clarify whether interference from irrelevant information might influence the obtained age differences in visual error detection.

Our findings suggest important practical considerations for older adults. For example, there are implications of decreased error and anomaly detection for aging drivers, who may fail to notice an “error” (e.g., a nonfunctioning stoplight; a car on the wrong side of the road), especially in an otherwise familiar scene (see [35] for evidence that older adults report “unexpected vehicles” as a major driving problem). There are also implications for older eyewitnesses, who may fail to accurately represent aspects of a crime scene because novel visual information (e.g., people with weapons) is introduced into an otherwise familiar scene (their neighborhood bank). Such practical applications indicate that further investigation is warranted to better understand the mechanisms underlying age differences in error detection, with the eventual goal of developing interventions to increase older adults' ability to accurately represent errors and other novel visual information.

## Figures and Tables

**Table 1:** Participant characteristics in Experiments  1 and 2 (SDs in parentheses).

	Young adults	Older adults
Experiment 1	*N* = 18 (14 females; 4 males)	*N* = 18 (10 females; 8 males)
Age in years	24.00 (4.04)	71.72 (5.96)
Years of education	14.89 (1.41)	14.71 (2.52)
Near vision test score	20/26 (7.52)	20/36 (16.08)

Experiment 2	*N* = 20 (15 females; 5 males)	*N* = 20 (11 females; 9 males)
Age in years	23.35 (3.62)	74.60 (7.09)
Years of education	15.13 (1.23)	14.63 (3.27)
Near vision test score	20/25 (5.62)	20/28 (6.18)

**Table 2:** Percent of errors detected, overall and separately for each stimulus picture in Experiments  1 and 2 (SDs in parentheses).

	Young adults	Older adults
Experiment 1		
Overall	81% (10%)	68% (15%)**
Picture 1	73% (12%)	60% (16%)**
Picture 2	87% (7%)	74% (15%)**
Picture 3	87% (14%)	73% (17%)*

Experiment 2		
Overall	85% (12%)	70% (17%)**
Picture 1	98% (8%)	93% (12%)
Picture 2	98% (7%)	76% (25%)
Picture 3	73% (26%)	55% (16%)
Picture 4	96% (11%)	70% (25%)
Picture 5	50% (0%)	45% (37%)
Picture 6	67% (30%)	53% (32%)

*indicates a significant age difference, *P* < 0.05; **indicates a significant age difference, *P* < 0.01. Individual pictures within Experiment 2 were not tested for age differences^2^.

## Endnotes

1. For more detailed discussion of the processes involved in error detection within the transmission deficit framework, see MacKay [36], MacKay and James [23], and Shafto [2].
2. There were too few errors per picture in this experiment to meaningfully run ANOVAs with picture as a variable, although the direction of the difference favored young adults for each picture (see Table 2). The variability of the magnitude of the age effect across pictures probably results from the restricted range of possible scores (there were as few as two errors per picture). However, we cannot rule out that this variability reflects important differences in the types of errors and anomalies or their arrangement within each picture, which should be followed up in future research.
